# Uses of Human Tissue Bioburden Data

**DOI:** 10.1111/tid.70264

**Published:** 2026-07-09

**Authors:** Melissa A. Greenwald, Eduardo Nunes

**Affiliations:** ^1^ Association for Advancing Tissue and Biologics Washington DC USA; ^2^ F. Edward Hebert School of Medicine Uniformed Services University of the Health Sciences Bethesda Maryland USA

**Keywords:** bioburden, preprocessing cultures, tissue for transplantation, tissue safety

Montedonico et al. [1] presented a study of pre‐processing bioburden culture results for tissues collected in the United States between 2019 and 2025. Aggregating this type of US tissue banking data is difficult to achieve for many reasons, and the effort and analysis for this study are commendable. The evaluation of these findings requires an understanding of the broader context of modern tissue recovery and processing, which merits being highlighted.

As indicated by the authors, cultures used to quantify and identify the type of organisms present on recovered tissues prior to processing (i.e., the bioburden), which comprise both intrinsic microbial flora from the donor and any contamination during recovery, are required by the Association for Advancing Tissue and Biologics (AATB) standards and recommended by Food and Drug Administration (FDA) guidance. It is also a fundamental requirement for process validation and monitoring (regardless of whether the processing method used is solely aseptic, includes liquid chemical sterilization, or includes ionizing irradiation). The bioburden must be known to ensure that any tissue going into a validated process can be reliably managed, otherwise there is the risk for communicable disease transmission.

Bioburden culture data are also collected since post‐recovery tissue is not presumed to be sterile. These culture data are used by individual tissue establishments for many purposes (Table 1), and they represent an important part of the overlapping layers of safety in tissue transplantation. These layers of safety include donor selection and evaluation, required infectious disease testing, additional infectious disease testing, processing (sometimes including sterilizing steps), careful tissue handling, and the receipt and careful investigation of possible adverse event reports.

**TABLE 1:** Uses of tissue bioburden data.

Organization‐level bioburden data	National‐level bioburden data
Inform the use of tissue Discard (individual tissues or all tissues from a donor) Aseptic processing Sterilization	Benchmarking
Donor evaluation Multiple preprocessing cultures positive for the same organism may be an indication of previously unidentified systemic infection	Starting point for new establishments looking to get a sense of whether their results are aligned/reasonable
Quality control/monitoring purposes Quality of recovery technique Possibility of contamination at recovery sites Unusual or unexpected organisms may require investigation Available to aid in investigation of adverse events	General awareness of microbiological trends in recovered human tissue intended for transplantation

Tissue for transplantation has ample opportunity for contamination or cross‐contamination due to the length of time and multiple steps between recovery and transplantation. Tissue is recovered from humans (all of whom have a microbiome and some of whom may have infections), is transported and stored at one or multiple processing facilities to await processing, undergoes varying number and types of processing steps, and may be shipped for specialty processing steps (e.g., irradiation) prior to release of tissue for distribution. Assessment of bioburden data from a donor, and often other aspects of the donor profile (e.g., donor age, cause of death), are used in combination to determine the appropriate processing method, if any, suitable for the tissue. For example, skin allografts often come from older donors and total joints often come from younger donors. While tissues with bioburden, including *Clostridium* or Group A *Streptococcus*, are required by AATB and recommended via guidance by FDA to be processed using a terminal sterilization process validated to a sterility assurance level (SAL) of 10^−6^, or be discarded [2, 3].

Ensuring the quality and safety of tissue allografts for transplantation begins with recovering tissues in a manner that contains as little bioburden as possible. However, these US tissue data provide an important reminder that it is unrealistic to expect no bioburden on recovered tissue. Processing is intended to mitigate microbial risks in order to best utilize the donated gift while providing safe tissue for transplantation. The manuscript authors mentioned the important limitations of MedWatch adverse event data review performed by the FDA's Tissue Safety Team, and we agree that reported events do not equate to a disease transmission event via tissues. The voluntary nature of adverse event reporting assumes a likelihood of under‐reporting of potential allograft infections. There are many confounders, including risks posed by the surgery and the patient's wound care, that can make it extraordinarily difficult to definitively ascertain the source of a post‐transplant infection [4]. The manuscript underscores the importance of clinicians maintaining an index of suspicion when considering the tissue allograft as a possible source of post‐tissue transplant infection and reporting those events to tissue processors for investigation. Reports where postoperative infections match up with the bioburden culture results would implicate the tissue as the source of infection and are infrequent, with a notable exception being that of Group A *Streptococcus* infection reported in 2003 [5].

When properly validated and executed, tissue processing is highly effective in providing safe tissue for transplantation. It is notable that the bacterial transmission events cited in the Montedonico manuscript, with the exception of *Mycobacterium tuberculosis* (MTB), are clustered in the 2000s. Indeed, those unfortunate events certainly informed needed improvements in tissue banking procedures. Since the FDA's 21 CFR Part 1271 regulations went into effect in 2005, and along with the evolution of AATB standards, significant improvements have been made in many areas of tissue banking, including the requirements for evaluating preprocessing (bioburden) cultures, process validation, more standardized donor screening and testing, as well as improvements in tissue processing methodology.

It should be noted that the testing methodology used in this study cannot detect MTB, and while sensitive and specific testing within a reasonable timeframe that would reliably detect MTB would be ideal, there are real challenges to implementing additional universal testing of all incoming tissues for MTB that are currently under review [6].

While these data may not be directly clinically relevant, having awareness of how tissues are handled, evaluated, and processed can help inform the investigation of a postoperative infection. There is no room for complacency in tissue transplantation—recipient safety is of the utmost importance. While preprocessing bioburden culture results reported need to be considered in context, the collection and continual monitoring of these data are vital to tissue safety.

## Author Contributions

**Melissa A. Greenwald**: conceptualization, Writing – original draft, writing – review and editing. **Eduardo Nunes**: conceptualization, writing – review and editing.
